# ECG changes associated with lithium intoxication – a study based on the lisie project

**DOI:** 10.1192/j.eurpsy.2021.333

**Published:** 2021-08-13

**Authors:** P. Truedson, K. Lindmark, M. Ström, M. Maripuu, U. Werneke

**Affiliations:** 1 Sunderby Research Unit, Umeå University, Deparment of clinical science, division of psychiatry, Luleå, Sweden; 2 Department Of Public Health And Clinical Medicine- Medicine, Umeå University, Umeå, Sweden; 3 Medical Intern, Piteå Hospital, Piteå, Sweden; 4 Department Of Clinical Sciences- Psychiatry, Umeå University, Umeå, Sweden

**Keywords:** ECG, Lithium intoxication

## Abstract

**Introduction:**

It currently remains unclear in how far supratherapeutic lithium serum concentrations can affect the cardiac conduction system. Prolonged QT interval, arrhythmias and cardiac death have all been anecdotally reported, but the systematic studies are few.

**Objectives:**

To examine ECG changes occurring with supratherapeutic lithium concentrations that have given rise to lithium toxicity.

**Methods:**

We examined all episodes of lithium intoxication defined as serum lithium level (≥ 1.5 mmol/L). We analyzed ECG before, during and after intoxication and recorded ECG changes. These, we then assessed according to type of intoxications, clinical and other pharmacological characteristics. The study is based on 20-year data (1997-2020) from the retrospective cohort study (LiSIE) in the Swedish region of Norrbotten.

**Results:**

Of 1101 patients treated with lithium, 77 patients had experienced lithium intoxications. 12 patients had more than one episode of intoxication, yielding 91 episodes. 39 had ECG available both as reference and during lithium intoxication. We found no statistically significant prolongation of the QTc interval during lithium intoxication, compared to respective reference ECG (p = 0.364). Heart rate during lithium intoxication was significantly lower, mean 73 beats/min (SD 16,8, range 43 - 112), compared to the reference ECG, mean 79 beats/min (SD 15,3, range 48-112; p = 0.006). No patient died. All findings were independent of whether an intoxication was acute or chronic.

**Conclusions:**

In our study, heart rate was significantly lower during episodes of intoxication. However, this decrease was of no clinical relevance in most cases. Lithium intoxication was not associated with prolonged QT time.

**Disclosure:**

M. Ott: scientific advisory board member of Astra Zeneca, Sweden. U. Werneke: received funding for educational activities on behalf of Norrbotten Region; Astra Zeneca, Eli Lilly, Janssen, Novartis, Otsuka/Lundbeck, Servier, Shire, Sunovion. Others: None

